# Ordered versus Non-Ordered Mesoporous CeO_2_-Based Systems for the Direct Synthesis of Dimethyl Carbonate from CO_2_

**DOI:** 10.3390/nano14181490

**Published:** 2024-09-13

**Authors:** Nicoletta Rusta, Fausto Secci, Valentina Mameli, Carla Cannas

**Affiliations:** 1 Department of Chemical and Geological Sciences, University of Cagliari, S.S. 554 bivio per Sestu, 09042 Monserrato, CA, Italy; n.rusta@studenti.unica.it (N.R.); fausto.secci@unica.it (F.S.); valentina.mameli@unica.it (V.M.); 2 Consorzio Interuniversitario Nazionale per la Scienza e Tecnologia dei Materiali (INSTM), Via Giuseppe Giusti 9, 50121 Firenze, FI, Italy

**Keywords:** CO_2_ utilization, dimethyl carbonate, ceria, mesoporous, nanocomposites, catalysis

## Abstract

In this work, non-ordered and ordered CeO_2_-based catalysts are proposed for CO_2_ conversion to dimethyl carbonate (DMC). Particularly, non-ordered mesoporous CeO_2_, consisting of small nanoparticles of about 8 nm, is compared with two highly porous (635–722 m^2^/g) ordered CeO_2_@SBA-15 nanocomposites obtained by two different impregnation strategies (a two-solvent impregnation method (TS) and a self-combustion (SC) method), with a final CeO_2_ loading of 10 wt%. Rietveld analyses on XRD data combined with TEM imaging evidence the influence of the impregnation strategy on the dispersion of the active phase as follows: nanoparticles of 8 nm for the TS composite vs. 3 nm for the SC composite. The catalytic results show comparable activities for the mesoporous ceria and the CeO_2_@SBA-15_SC nanocomposite, while a lower DMC yield is found for the CeO_2_@SBA-15_TS nanocomposite. This finding can presumably be ascribed to a partial obstruction of the pores by the CeO_2_ nanoparticles in the case of the TS composite, leading to a reduced accessibility of the active phase. On the other hand, in the case of the SC composite, where the CeO_2_ particle size is much lower than the pore size, there is an improved accessibility of the active phase to the molecules of the reactants.

## 1. Introduction

Due to the critical consequences of global warming and climate change caused by the drastic increase in the emissions of anthropogenic greenhouse gases into the atmosphere, one of the main current challenges is the reduction in CO_2_, the most problematic greenhouse gas. In particular, one of the ways to decrease the CO_2_ concentration is by capturing [1,2,3] and valorizing it to obtain value-added products (Carbon Capture and Utilization technologies, CCU) like chemicals (e.g., formic acid [4] and dimethyl carbonate [5,6]) and fuels (e.g., methane [7,8], methanol [4,9], and dimethyl ether [4,10,11,12,13]). In this context, the direct synthesis of dimethyl carbonate (DMC) represents a good opportunity to chemically convert CO_2_ into a valuable solvent applied in different fields, e.g., energy storage (Li-ion batteries) and industrial chemistry (polycarbonates production) [5,6,14]. DMC is traditionally synthesized by many different routes, like the phosgene method, the oxidative carbonylation of methanol, the gas-phase carbonylation of methyl nitrite, the transesterification method, and the alcoholysis of urea. All these methods, however, involve the use of toxic reagents and catalysts or the production of undesired byproducts [5,6,15]. On the other hand, the production of DMC from CO_2_ and methanol is not only an environmentally friendly route, since it involves the valorization of CO_2_ as a raw material, but also does not require the use of highly toxic gas-phase reagents or harmful catalysts. Furthermore, this reaction only produces water as a byproduct and requires low amounts of energy and relatively low-cost equipment [5,6,16]. However, due to the chemical inertness of CO_2_, the reaction needs to be catalyzed; in particular, ceria-based systems are among the most proposed metal oxide catalysts due to their chemical stability, redox properties, and high catalytic activity [6,16,17,18,19,20,21,22]. However, cerium is an expensive and critical raw material, making it essential to reduce its use in catalysts to lower both the environmental impact and the cost of the process. For these reasons, porous CeO_2_ and, in particular, mesoporous ceria or nanoceria incorporated into mesostructures are promising systems for maximizing the surface area and favoring the diffusion of reactants and products [23]. Mesoporous CeO_2_ has been synthesized in different ways, such as hard template and soft template processes, the sol–gel route, hydrothermal/solvothermal approaches, and precipitation [23]. Hard templating consists of the use of an ordered mesoporous structure (usually silica or carbon) as a template; this template is impregnated with the precursor species of CeO_2_ and, after CeO_2_ is formed, the template is removed either by etching (silica) or by combustion (carbon). This approach allows for obtaining ordered crystalline mesoporous ceria with a uniform and adjustable pore size and high thermal stability, but the synthesis processes are often complex, expensive (due to the use of a mesostructured template as a sacrificial material), and the template (particularly silica) is often not easy to remove [23,24,25]. On the other hand, the soft template method involves the use of organic “soft” templating species, like block copolymers, polymers, or smaller organic molecules; these templates are cheaper and easier to remove than hard templates, however, the obtained mesoporous CeO_2_ has a disordered pore structure, a broader pore size distribution, and a lower thermal and mechanical stability [23,26,27,28]. Sol–gel methods also often involve the use of organic soft templates, and their use is combined with a sol–gel approach consisting of a hydrolysis reaction, subsequent condensation, and a final annealing of the obtained material. These syntheses are cheap and sustainable, but also lead to the formation of non-ordered porous structures, often associated with low surface areas [23,29,30,31]. Hydrothermal/solvothermal methods rely on the use of a sealed vessel to perform high-temperature treatments; the use of different temperatures allows for modifying the shape of the nanoparticles and their surface areas, but an ordered mesoporous structure is often not obtainable [23,32,33,34]. Finally, precipitation methods involve a precipitation reaction between a precursor and a precipitating agent; these approaches, often assisted by the use of soft templating agents, are easy and straightforward, allowing for reaching high surface areas but with a non-ordered porous structure [23,35].

Due to its high exposed surface area, several applications have been reported for mesoporous ceria, like catalysis, photocatalysis, water remediation, air purification, the degradation of organic pollutants, drug delivery, fuel cells, and sensors. Regarding catalysis, however, only a small number of papers have reported the use of mesoporous CeO_2_ as catalyst for the synthesis of DMC from CO_2_ and methanol [17,36,37].

Another strategy for designing easily accessible CeO_2_-based systems is to develop mesostructured composites incorporating CeO_2_ into the channels of inert mesostructures as mesostructured silica (SBA-15 and MCM-41). Within this framework, two-step [38,39,40,41,42,43] and one-step [38,44,45] procedures have been proposed. In two-step methods, CeO_2_ nanoparticles are incorporated inside the pores of a pre-synthesized support by post-synthesis methods; on the other hand, in the one-step approach, the support is synthesized, usually from alkoxide precursors, together with the CeO_2_ particles, by introducing both Si and the Ce precursors in the same reaction batch. Regarding two-step methods, different impregnation strategies have been used to functionalize the silica walls, mainly based on the use of aqueous solutions [40,42,43]. Indeed, the silanol groups at the surface render the silica hydrophilic and accessible to aqueous solutions containing the cerium precursors. Besides impregnation approaches involving the use of aqueous solutions, another method, called the molten nitrate method, based on an apolar solvent [39], has been proposed; it consists of the dispersion of the support and cerium nitrate in an apolar solvent (toluene), which is then heated beyond the melting point of cerium nitrate, allowing for its introduction into the pores of the support. Furthermore, solid-state approaches, based on the grinding of cerium nitrate with the support followed by a thermal treatment to induce the decomposition of nitrates [38,41], have been proposed.

Among the cited papers, the only one dealing with the development of CeO_2_@SBA-15 composites for the synthesis of DMC from CO_2_ and methanol used a slurry impregnation approach, based on the insertion of an aqueous solution of cerium nitrate into the mesopores of the support, followed by the evaporation of water and a final thermal decomposition of the cerium nitrate to CeO_2_ [40].

Among the various impregnation methods reported in the literature, a promising strategy is based on the use of two solvents, as follows: a polar solvent, like water, and an apolar solvent, to favor the loading of metal precursor solutions into the hydrophilic pores. This method was efficiently used to develop regenerable and efficient sorbents for H_2_S removal based on ZnO and Fe_2_O_3_ in supports like SBA-15 [46,47], MCM-41 [48], and MCM-48 [48], and was demonstrated to be more efficient than the conventional water-based incipient impregnation. To allow for the complete incorporation of the metal oxide precursors inside the pores of the support, this method relies on using an amount of aqueous solution corresponding to the pore volume of the support.

Another rarely used strategy involves the combination of an impregnation route with a self-combustion reaction. This approach consists of the impregnation of the support with an aqueous solution of the metal nitrates and a reducing agent; after the evaporation of the water, the self-combustion reaction (i.e., a redox reaction between the oxidant nitrates and the reducing agent) is ignited with a thermal treatment, leading to the formation of the metal oxides. The self-combustion method has been widely reported and is of particular interest, since it allows for obtaining various supported and unsupported nano-sized metal oxides [49,50,51,52,53] due to the presence of a reducing agent together with the nitrate (oxidizer) in the reaction environment. Furthermore, this method usually relies on the use of only water as a solvent and on cheap and environmentally friendly reducing agents (citric acid and glycine), resulting in being a cheap and green approach. However, regarding its use in combination with an impregnation approach, only a few instances have been reported, focused on the obtainment of Cu-based nanocomposites on SBA-15 or mesostructured γ-Al_2_O_3_ [54,55].

In this work, we present a non-ordered mesoporous CeO_2_ catalyst; for its synthesis, a precipitation approach assisted by soft templating is chosen, due to its simplicity, quickness, and cheapness. With the aim of investigating both the possibility of reducing the amount of active phase and the effect of an ordered mesoporous structure, the mesoporous CeO_2_ catalyst is compared with two different CeO_2_@SBA-15 composites. In this context, SBA-15 is chosen as support due to its large pore size, which allows it to easily host an active phase in the form of nanoparticles, compared to other mesostructures with smaller pores (MCM-41). Since the impregnation strategy has been demonstrated to strongly influence the dispersion of the active phase into the porous support, the particle size, and the crystallinity, all critical features for catalytic activity, we focus on two impregnation routes, namely two-solvent and self-combustion impregnation. These strategies, indeed, as mentioned above, prove to be able to efficiently disperse several metal oxides onto different mesostructured siliceous supports (i.e., SBA-15, MCM-41, and MCM-48) [46,47,48,54,55]. Furthermore, to the best of our knowledge, these impregnation methods, rarely reported in the literature, have never been used to synthesize CeO_2_-based composites.

## 2. Materials and Methods

**Chemicals.** Cerium(III) nitrate hexahydrate (99.5%, Acros Organics, Geel, Belgium) was used in all CeO_2_ syntheses. NaOH (pellets, Sigma Aldrich, St. Louis, MO, USA) and cetyltrimethyl ammonium bromide (CTAB) (98%, Sigma-Aldrich) were used in the soft-template surfactant-assisted precipitation synthesis. Pluronic P123 (average number average molecular weight ≈ 5800, Sigma-Aldrich), HCl (37%, VWR Chemicals, Radnor, PA, USA), and TEOS (98%, Acros Organics) were used in the synthesis of the SBA-15 support. Hexane (97%, VWR Chemicals) and bi-distilled water were used in the two-solvent impregnation synthesis. Citric acid (99.5%, Aldrich) was used in the impregnation combined with self-combustion synthesis. All reagents were used as received, without further purification.

**Synthesis of mesoporous CeO_2_ (CeO_2__Meso).** Mesoporous ceria was synthesized using a soft-template surfactant-assisted precipitation method with cetyltrimethyl ammonium bromide (CTAB) as the templating agent, following the procedure reported in [35]. Typically, 1 g of CTAB was added to a cerium nitrate solution (2.17 g of Ce(NO_3_)_3_·6H_2_O in 200 mL) in a 500 mL round-bottom flask at room temperature and stirred gently. Then, a NaOH solution (1 g in 150 mL of distilled water) was added dropwise with continuous stirring at 150 rpm. After this addition was complete, the flask was sealed, and the mixture was maintained under constant stirring for 24 h. Following thermal aging at 90 °C for 3 h, the pale-yellow precipitate was filtered and washed twice with 200 mL of hot distilled water (80 °C). The sample was dried in a static oven at 100 °C for 6 h and then calcined at 450 °C for 4 h (heating rate of 5 °C per minute).

**Synthesis of the SBA-15 support (SBA-15).** The synthesis of the SBA-15 support was carried out by adapting the procedure reported by Zhao et al. [56,57]. Typically, 4 g of Pluronic P123 was dissolved in 120 g of HCl 2 M and 30 g of bi-distilled water in an Erlenmeyer flask by stirring at 600 RPM and at 35 °C in a water bath for 24 h. Then, the stirring was decreased to 100 RPM and maintained overnight. Then, 9 g of TEOS was added dropwise and the stirring was maintained for other 24 h at 35 °C. The resulting suspension was then put into a sealed Teflon-lined autoclave and heated at 100 °C in static conditions for other 24 h. The product was subsequently filtered, washed with warm distilled water (75–80 °C), and dried at 35 °C for 24 h. The obtained powder was finally calcined at 550 °C for 6 h with a ramp of 5 °C min^−1^.

**Synthesis of CeO_2_@SBA-15_TS.** For the synthesis of the CeO_2_@SBA-15_TS composite, SBA-15 was impregnated with 10% in weight of CeO_2_, adapting the two-solvent approach reported in [46,47,48]. In a typical synthesis, the support was firstly dried at 120 °C overnight to remove the adsorbed water; 0.5 g of the support was then submerged in 10 mL of hexane in a beaker that was then covered with a watch glass and maintained under stirring at 300 RPM for 2 h. The stirring was increased to 400 RPM and 0.57 mL of a 0.56 mM Ce(NO_3_)_3_ 6H_2_O aqueous solution was added dropwise. After 2 h, the watch glass was removed from the beaker and the temperature was set to 80 °C to let the hexane evaporate; when the evaporation was almost complete, the beaker was put into an oven at 80 °C overnight. Eventually, the obtained powder was calcined at 500 °C for 2 h with a 2 °C min^−1^ ramp.

**Synthesis of CeO_2_@SBA-15_SC.** For the synthesis of the CeO_2_@SBA-15_SC composite, SBA-15 was impregnated with 10% in weight of CeO_2_, adapting the self-combustion approach reported in [54,55]. Typically, 5.7 mL of a 5.6 × 10^−2^ mM aqueous solution of Ce(NO_3_)_3_ 6H_2_O containing citric acid, with a citric acid/Ce molar ratio of 1:1, was dropped onto 0.5 g of the support, after previous drying at 120 °C, in a beaker under vigorous stirring until a viscous paste was obtained. Then, it was sonicated for 5 min and submitted to a 300 °C treatment for 1 h in a pre-heated oven to induce the self-combustion reaction between the nitrates (oxidizing agents) and citric acid (reducing agent).

**Characterization techniques.** Wide-angle X-ray diffraction (WA-XRD) patterns were acquired in the 2θ range of 10–100° using a PANalytical X’pert Pro (Malvern PANalytical, Malvern, UK) equipped with a Cu Kα source (1.5418 Å). Small-angle X-ray diffraction (SA-XRD) patterns were acquired in the 2θ range of 0.7–3° using a Seifert X3000 instrument (Seifert, Radevormwald, Germany) equipped with a Cu Kα source. The hexagonal lattice parameter of the mesostructured samples was calculated using the equation $a_{0}=\frac{{2d}_{100}}{\sqrt{3}}$. A Rietveld analysis was performed with the software MAUD version number 2.997. LaB_6_ from NIST was used as a reference material to determine the instrumental parameters. The CIF structure used for the refinement was 1562989. The simulation of aluminum silica glass was carried out by the means of the Le Bail model [58,59].

Nitrogen physisorption isotherms were acquired at −196 °C using a 3Flex physisorption/chemisorption analyzer provided by Micromeritics. All samples were treated under vacuum at 250 °C (heating ramp, 1 °C/min) for 12 h before the analysis. The Brunauer–Emmett–Teller (BET) specific surface area (SA) was calculated from the adsorption branch in the 0.04–0.3 P/P^0^ interval. The total pore volume (V_p_) was determined at P/P^0^ = 0.99, and the mean pore diameter (D_p_) was extrapolated by applying the Barrett–Joyner–Halenda (BJH) model to the desorption data for all samples. The pore wall thickness (T_w_) was calculated using the formula $\;T_{w}=a_{0}-D_{p}$.

Transmission Electron Microscopy (TEM) images and Energy-Dispersive X-ray (EDX) characterization were carried out using a JEOL JEM 1400-PLUS microscope (JEOL, Akishima, Tokyo, Japan) operating at an accelerating voltage of 120 kV. The samples were first finely ground and dispersed in ethanol by an ultrasound treatment. The obtained suspensions were deposited onto 200-mesh carbon-coated copper grids.

UV–Vis–NIR solid-state absorbance spectra were collected (applying baseline corrections using Teflon as a reference) by a Jasco V-750 spectrophotometer (Tokyo, Japan) in the 200–800 nm range with a spectral bandwidth of 5 nm, a data interval of 1 nm, and a data pitch of 0.1 nm.

Thermogravimetric Analysis (TGA) was performed using a PerkinElmer STA 6000 (Waltham, MA, USA) in the 25–900 °C range, with a heating rate of 10 °C/min under a 40 mL/min O_2_ flow.

**Catalytic tests.** The catalytic tests for DMC synthesis were performed in batch conditions under magnetic stirring, using a 100 mL high-pressure reactor manufactured by Berghof (BR-100) (Eningen, Germany). For each test, 0.250 g of the catalyst, previously dried at 120 °C overnight, was put into the reactor together with 10 mL of liquid methanol (≥99.8%); the reactor was then purged three times with CO_2_ in order to remove air, subsequently pressurized at 5.0 MPa with CO_2_ (99.9%), and heated to 150 °C with a heating rate of 2 °C min^−1^. After 3 h of reaction, the reactor was cooled down to room temperature and the catalyst was recovered by centrifugation. The reaction products were analyzed using a gas chromatograph equipped with a flame ion detector (Agilent Technologies 6890N GC-FID, Santa Clara, CA, USA) and a capillary column (Zebron ZB-WAX, 30 m × 0.25 mm × 0.25 μm, Anaheim, CA, USA), using helium as a carrier gas with a flow rate of 1 mL min^−1^. 1-Propanol (≥99.8%) was added to dimethyl carbonate in methanol as an internal standard to quantitatively analyze the products using the calibration curve method. The DMC yield (mmol g_cat_^−1^) was estimated using Equation (1), as follows:

(1) $$DMC\;yield\left(mmolg_{cat}^{-1}\right)=\frac{DMC\;formation(mmol)}{Catalyst(g)}$$

## 3. Results and Discussion

**Figure 1:**
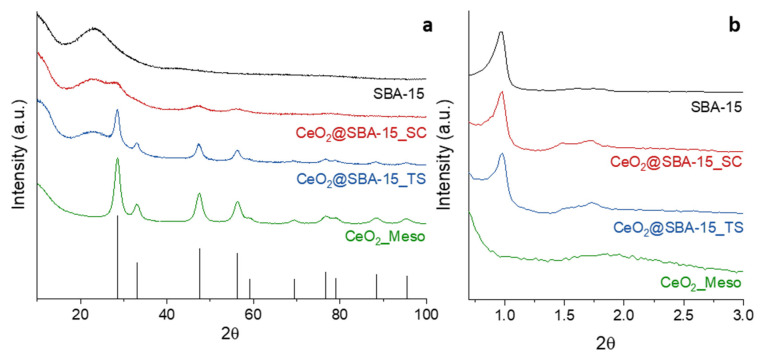
WA-XRD (**a**) and SA-XRD (**b**) patterns of all the samples.

The wide-angle XRD patterns (WA-XRD) of the investigated systems are shown in Figure 1a. The CeO_2__Meso sample shows the diffraction peaks attributable to cubic CeO_2_ (PDF card 00-034-0394), and no other phases were detected. The Rietveld analysis (Figure S1) points out a mean crystallite size of 7.9 ± 0.1 nm. The same signals from CeO_2_ are observed in the XRD pattern of the CeO_2_@SBA-15_TS, together with a broad band with a maximum located at a 2θ value of about 23°, ascribed to the amorphous silica of the SBA-15 support, the pattern of which has been reported for reference. Interestingly, also in this case, the Rietveld analysis (Figure S2) gives a mean crystallite size of 7.9 ± 0.1 nm, allowing for a direct comparison with the unsupported CeO_2__Meso system. Differently from that observed with the TS composite, the CeO_2_@SBA-15_SC sample shows very broad signals at 2θ values of about 28.5°, 47.5°, and 56.4°, corresponding to the three most intense crystalline reflections of cubic CeO_2_, respectively, (111), (220), and (311); also in this case, the amorphous band attributed to the support is visible. The Rietveld analysis (Figure S3) points out a mean crystallite size of 2.6 ± 0.1 nm, rather smaller than the pore size of the support (Table 1). This finding suggests that the CeO_2_ active phase was dispersed by the impregnation in the form of ultra-small nanoparticles, presumably inside the mesopores of the support. This result can likely be attributed to the rapid propagation of self-combustion reactions, which leads to a very fast conversion of metal nitrates in metal oxides. On the other hand, the thermal decomposition of a nitrate to an oxide, which takes place during the functionalization with TS impregnation, is presumably slower, being driven merely by the temperature rise. Thus, it can be inferred that, during the fast self-combustion reaction, the metal oxide particles did not have the time to grow as much as they could during nitrate decomposition, leading to the formation of smaller nanoparticles.

The small-angle XRD patterns (SA-XRD, Figure 1b) of CeO_2__Meso only show an extremely broad band centered at a 2θ value of about 1.8°, suggesting a possible disordered mesoporosity. On the other hand, the patterns of the two composites and their support show a main signal (100) located at a 2θ value of 0.98° and two lower signals at higher 2θ values. These signals are attributable to a hexagonal mesoporous arrangement (p6mm), typical of the SBA-15 structure, indicating that the ordered mesoporous arrangement was maintained after the functionalization process. It can be noticed that the positions of the main mesostructure peaks (100) are the same (Table 1) for both the support and the two composites, presumably indicating that the lattice parameter of the mesopore arrangement did not change significantly with the impregnation.

**Figure 2:**
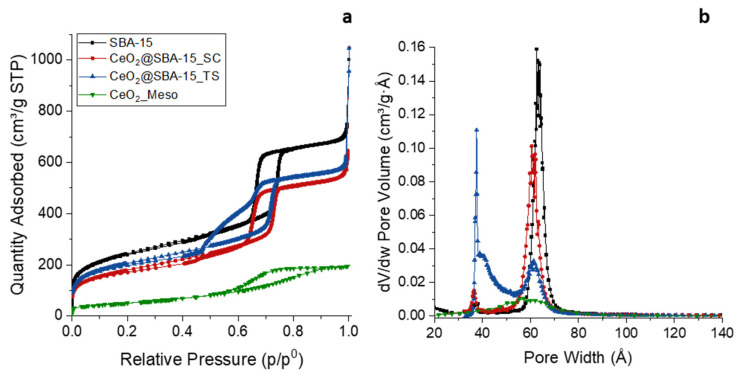
Nitrogen physisorption isotherms (**a**) and BJH pore size distributions (**b**) of all the samples.

All nitrogen physisorption isotherms (Figure 2a) can be described as type IV, typical of mesoporous samples, since they all feature a capillary condensation branch. The physisorption isotherms of the CeO_2__Meso sample, despite being attributable to a mesoporous material (type IV), show a significantly wide hysteresis cycle with capillary condensation branches that are not very steep, indicating the presence of a disordered mesoporosity, as already suggested by the SA-XRD analysis. On the other hand, the isotherms of the two composites show a very steep capillary condensation adsorption branch, comparable to that of the support (H1 hysteresis cycle) reported for reference, indicating that the mesoporous order was maintained after the incorporation of CeO_2_. Furthermore, the adsorption branch of the support has about the same value of relative pressure as the two composites (about 0.75), indicating that the functionalization did not cause a significant narrowing of the pores. In the TS composite, it can be noticed that the capillary condensation desorption branch is far less steep and presents a double concavity, presumably indicating a partial obstruction of the mesopores by the relatively large CeO_2_ nanoparticles, which causes the formation of inkbottle mesopores (mesopores with a narrow opening) and, consequently, a slower emptying of the pores during the desorption [46,60,61]. As expected, a decrease in terms of surface area and pore volume is observed for both the composite, compared to the support (Table 1), ascribed to the functionalization with the active phase.

The BJH plot (Figure 2b) of CeO_2__Meso indicates an extremely wide pore size distribution with a maximum located at 5.8 nm, typical of a non-ordered mesoporous sample. Conversely, the BJH plot of the SC composite and the SBA-15 support show narrow pore size distributions with a similar width and mean pore size (6.1 nm for CeO_2_@SBA-15_SC and 6.3 nm for SBA-15), indicating, as already suggested by the SA-XRD analysis and the physisorption isotherms, that the impregnation process did not cause either a significant decrease in the mean pore diameter nor a loss of mesoporous order in terms of pore size distribution; also, the wall thickness does not show a significant change (Table 1). The BJH plot of the TS composite, on the other hand, shows a wide bimodal distribution, with a maximum at 6.1 nm, close to the original value of the pore size of the support, and another maximum at about 4 nm, reinforcing the hypothesis of a partial pore obstruction inferred by the observation of the desorption capillary condensation branch. Particularly, the maximum located at 6.1 nm is attributable to unoccupied pores, and the one at 4 nm is ascribed to the inkbottle mesopores formed by the incorporation of the CeO_2_ nanoparticles [46,60,61]. The maximum at 6.1 nm was used to calculate the wall thickness that, as observed for the SC composite, does not show significant differences with that of the support (Table 1).

**Figure 3:**
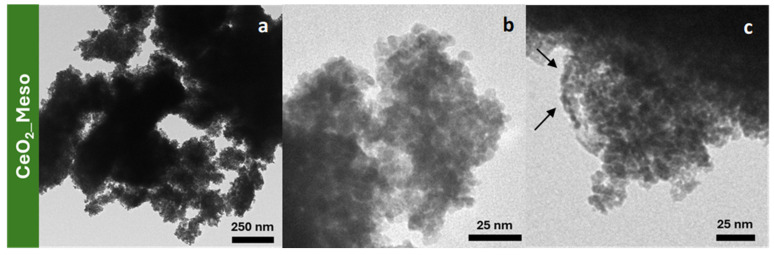
TEM micrographs of CeO_2__Meso (**a**–**c**).

The TEM micrographs of the samples CeO_2__Meso (Figure 3) show a material consisting of large aggregates mainly comprising nanoparticles of a spheroidal shape (with a size of 5–8 nm, in agreement both with the mean crystallite size pointed out by the Rietveld analysis and with the data reported in the literature for the same synthesis process [35]), along with a minor contribution of elongated ones, some of which consist of chains of spheroidal nanoparticles (indicated by arrows). This finding justifies the mesoporous nature of this system, which is ascribed to a disordered worm-like interparticle porosity.

**Figure 4:**
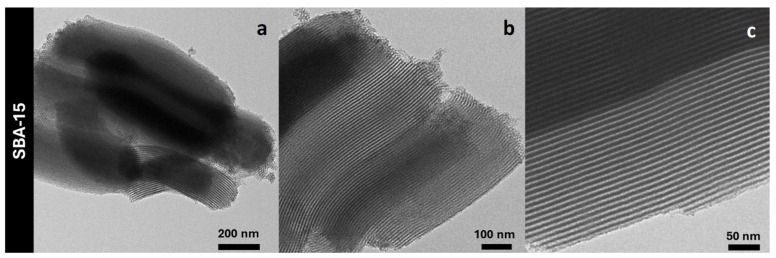
TEM micrographs of the SBA-15 support (**a**–**c**).

TEM imaging of the bare SBA-15 support (Figure 4) clearly shows the presence of an ordered mesoporous structure consisting of hexagonally-arranged parallel channels, in agreement with what has been observed in the literature for SBA-15 materials [56].

**Figure 5:**
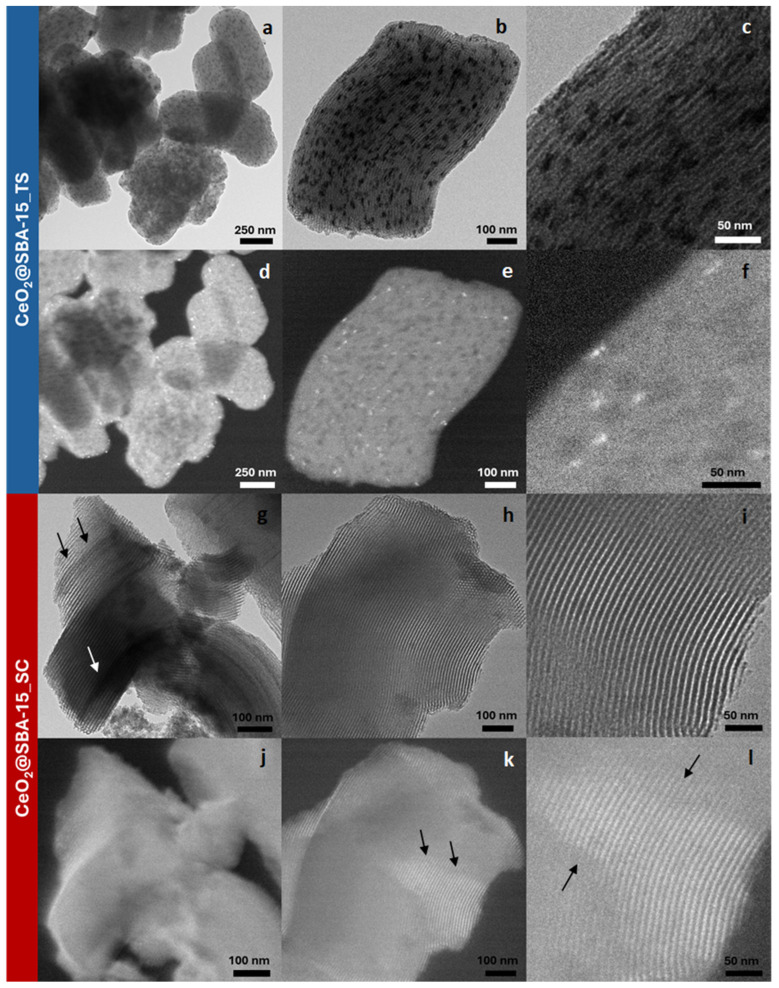
Bright-field (**a**–**c**,**g**–**i**) and dark-field (**d**–**f**,**j**–**l**) micrographs of CeO_2_@SBA-15_TS (**a**–**f**) and CeO_2_@SBA-15_SC (**g**–**l**).

From the TEM micrographs of the CeO_2_@SBA-15_TS nanocomposite (Figure 5a–c), it can be observed that the impregnation process led to the formation of elongated CeO_2_ nanoparticles (the darker spots visible in the micrographs) with a width of about that of the mesopore size (6–7 nm) and a variable length. The dark-field TEM imaging (Figure 5d–f) confirmed the crystalline nature of these particles, as they appear as bright spots. These results are in agreement with the Rietveld analysis, which points out a mean crystallite size of 7.9 nm; this size, indeed, despite being larger than the mean pore diameter of the support (6.3 nm), is attributable to the elongated form of the particles. Therefore, considering their peculiar, elongated shape (always oriented in the same direction of the mesochannels), their size (comparable with the mean pore diameter) and the fact that, at high magnification, the walls of the mesochannels are still visible, despite the presence of these particles, it can be assumed that CeO_2_ is incorporated inside the mesopores. This finding is in agreement with the nitrogen physisorption data, which show a capillary condensation desorption branch less steep that that of the support, presumably due to the partial obstruction of the pores by the CeO_2_ nanoparticles.

On the other hand, the CeO_2_@SBA-15_SC nanocomposite does not show, in the bright-field TEM micrographs (Figure 5g–i), any dark spot ascribable to nanoparticles of the active phase. In some zones, however, the mesochannels present a darker color for their whole length (see arrows in Figure 5g), presumably indicating an incorporation of CeO_2_ inside the pores in a more highly dispersed form (smaller nanoparticles). This assumption is confirmed by the dark-field TEM imaging (Figure 5j–l), which clearly points out the fine functionalization of the mesopores, indicated by the fact that the mesochannels appear as bright due to the presence of finely distributed ultra-small CeO_2_ crystalline nanoparticles inside them. From these observations, it can be assumed that the active phase was dispersed in form of very small nanoparticles, but some zones/mesochannels of the support present a higher loading of active phase than others, indicated by a higher contrast (darker zones) in the bright-field micrographs (Figure 5g), as well as by brighter zones in the dark-field micrographs, as evidenced by arrows in (Figure 5k,l).

**Figure 6:**
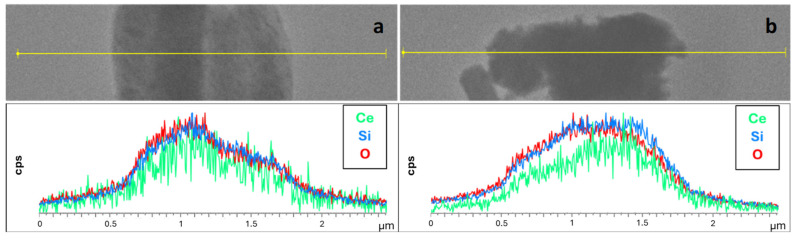
Line profile EDX analyses on CeO_2_@SBA-15_TS (**a**) and CeO_2_@SBA-15_SC (**b**). The data for Ce, Si, and O have been normalized.

The line-profile EDX analysis on CeO_2_@SBA-15_TS (Figure 6) shows an overall homogeneity in the distribution of the atomic species attributable to the support and the active phase (Si and Ce, respectively), indicating that all the CeO_2_ has presumably been properly dispersed inside the pores, with no segregation of CeO_2_ particles outside the pores. Furthermore, five EDX spectra were acquired in five different regions (Table S1), and all of them point out a CeO_2_ weight percentage between 9.5 wt% and 12.1 wt%, with a mean value of 11(±1) wt%, in very good agreement with the theoretical value of 10 wt% and with a low standard deviation.

The low homogeneity of functionalization with the active phase of the CeO_2_@SBA-15_SC composite, suggested by TEM imaging, is also supported by the line-profile EDX analysis (Figure 6 and Figure S6) which, for CeO_2_@SBA-15_SC points out a less homogeneous distribution of Ce and Si throughout the material, compared to CeO_2_@SBA-15_TS. The EDX spectra, acquired in thirteen different regions (Table S2), indicate the same average CeO_2_ wt% loading shown by the TS composite (11%), but with a significantly higher standard deviation (6%), indicating that the total CeO_2_ loading is the same for the two composites, but CeO_2_@SBA-15_SC has more local inhomogeneities. It can be presumed that these inhomogeneities in the functionalization are a consequence of the rapid metal nitrate–metal oxide conversion typical of self-combustion reactions mentioned before. It can, thus, be inferred that this rapid transition leads to an almost instantaneous formation of the oxide species, which do not have the time to equally distribute inside all mesochannels of the support.

Further characterization, performed by UV-Vis and TGA, is reported in the supplementary information (Figures S4 and S5).

**Figure 7:**
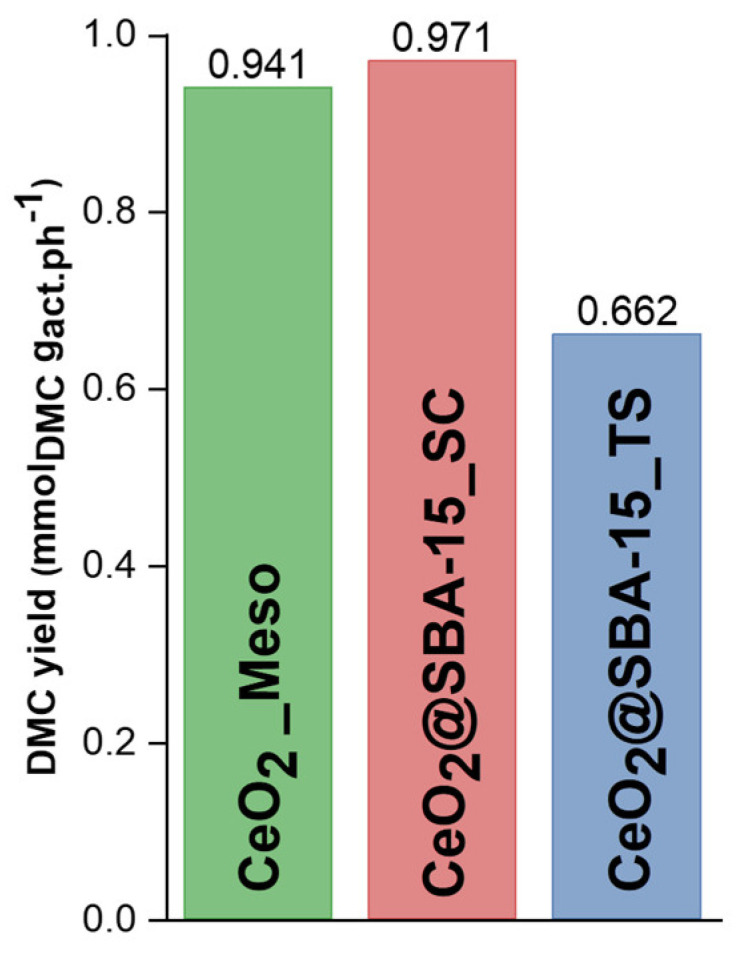
DMC yield (mmol/g_act_._ph_) of the catalysts.

The catalytic tests (Table 2) evidence that, as expected, the CeO_2__Meso sample shows a better performance in terms of DMC yield (0.941 mmol/g_cat_); this result can be ascribed to the fact that this catalyst is composed of pure CeO_2_, which is the active phase of the reaction. On the other hand, the two composites show lower values of the DMC yield (0.097 mmol/g_cat_ for CeO_2_@SBA-15_SC and 0.066 mmol/g_cat_ for CeO_2_@SBA-15_TS) due to the significantly lower amount of the active phase contained in these systems (10% in weight) compared to the CeO_2__Meso sample. However, it is important to point out that, considering the DMC yield expressed as a function of the amount of the active phase (Table 2; Figure 7), the CeO_2_@SBA-15_SC sample shows similar performances (0.971 mmol/g_act_._ph_.) to the CeO_2__Meso sample (0.941 mmol/g_act_._ph_.). The catalytic performances are normalized as a function of a CeO_2_ loading of 10%, considering that the synthesis methods used for the preparation of the composites do not involve any separation step. Thus, all the Ce precursors used for the synthesis remain as CeO_2_ in the final product, implying that the nominal and experimental CeO_2_ loading correspond. On the other hand, the CeO_2_@SBA-15_TS composite shows weaker performances (0.662 mmol/g_act_._ph_.). Considering that all the catalysts show similar performances (0.7–1 mmol/g_act_._ph_.), if normalized for the amount of the active phase, we can presume that the involved reaction mechanism is the same for all the catalysts and corresponds to the most widely accepted mechanism [62,63,64,65], involving the formation of the carboxy methoxide intermediate and its subsequent reaction with the methoxy species, formed by the dissociation of another methanol molecule.

The differences in terms of performance between the two nanocomposites with the same loading are likely to be ascribed to the different dispersions of the active phase deriving from the different impregnation methods used for the synthesis. Specifically, the two-solvent approach is based on the idea that the dispersion of a hydrophilic silica into an apolar solvent should favor the diffusion of a small volume of Ce-containing aqueous solution (corresponding to the pore volume of the support) inside the pores with hydrophilic walls during the evaporation of the apolar solvent. CeO_2_ is then obtained by the thermal decomposition of cerium nitrate incorporated into the pores. On the other hand, the impregnation route involving a self-combustion reaction relies on the use of a single solvent (water). The diffusion of the Ce-based solution inside the mesopores is, also in this case, favored by the evaporation of the solvent. Ceria, in this case, is obtained once again by a thermal treatment, but involving a fast exothermic redox reaction between the nitrate ions (oxidizers) and a reducing agent (citric acid). The higher homogeneity of dispersion in the case of the TS process is probably due to the slow evaporation of the high amount of apolar solvent, allowing for the gradual diffusion of the low amount of aqueous phase into the pores. The CeO_2_ nanoparticles reach the physical limit of the pore size (about 7 nm) as a consequence of the slow thermal decomposition of the ceria nitrate precursor, which gives the nanoparticles the time to grow inside the pores, leading to a partial pore obstruction. On the other hand, the lower degree of homogeneity shown by the SC composite can be likely ascribed to the use of a single solvent (water) with a volume about ten times higher than the pore volume of the support and also to the fast self-propagating redox reaction, which furthermore hampers the growth of the nanoparticles inside the pores, justifying the formation of very small ceria particles. From the catalytic results, it can be inferred that, rather than the homogeneity of the dispersion, the key factor to obtain better performances seems to be the accessibility of the active phase improved by the lower nanoparticle size, leading to a higher diffusion of reactants and products.

The acquired results are in agreement with the data reported in [66] for the theoretical value of yield (mol%) of DMC for this reaction under the investigated conditions of temperature, pressure, and feed ratio. However, Pu et al. [40] reported much better results (0.15 mmol/g_cat_) for a similar composite catalyst (CeO_2_/SBA-15), probably due to significantly different experimental conditions, like a higher CeO_2_ weight loading (12.3%), a lower temperature (130 °C), a higher reaction time (10 h), a smaller reactor volume (50 mL), and a greater amount of catalyst (500 mg).

## 4. Conclusions

In this work, a study on different non-ordered and ordered mesoporous CeO_2_-based catalysts for the CO_2_ conversion to DMC is presented. Particularly, a non-ordered mesoporous catalyst, consisting of pure CeO_2_ (CeO_2__Meso), is compared with two composites obtained by dispersing CeO_2_, with a 10 wt% loading, on an ordered mesoporous siliceous support (SBA-15), with the aim of maximizing the catalytic performance with a low amount of active phase. The two composites are obtained by functionalizing the support with two different impregnation strategies: a two-solvent impregnation (TS) method and an impregnation method combined with a self-combustion reaction (SC). The combination of XRD, nitrogen physisorption, and TEM characterization points out that, for the composite obtained with the TS strategy (CeO_2_@SBA-15_TS), the impregnation led to the formation of CeO_2_ nanoparticles of about 8 nm located inside the mesopores. On the other hand, the composite obtained with the SC method (CeO_2_@SBA-15_SC) features significantly smaller CeO_2_ nanoparticles (about 3 nm), also incorporated inside the pores of the mesostructured support. The study of the catalytic performances shows how CeO_2__Meso, consisting of pure CeO_2_, features the best performances; the composites, on the other hand, show worse performances, due to their lower amount of active phase (10%). By normalizing the catalytic activity as a function of the active phase of the catalyst, however, it can be noticed how the CeO_2_@SBA-15_SC composite shows similar performance (DMC yield = 0.971 mmol/g_act_._ph_.) to CeO_2__Meso (DMC yield = 0.941 mmol/g_act_._ph_.), presumably due to the fine dispersion of the active phase throughout the mesostructured matrix, leading to a high exposed area of the active phase. This assumption is also supported by the fact that the other composite, obtained by the TS approach and featuring larger CeO_2_ nanoparticles, with a possible pore obstruction, shows a lower catalytic activity (0.662 mmol/g_act_._ph_.). The combination of an impregnation strategy with a self-combustion reaction, thus, resulted to be the most promising method to obtain supported catalysts with a highly dispersed active phase, leading to an improvement in catalytic performances over the composite obtained by the two-solvent approach. Future studies will focus on the optimization of this approach by studying different reducing agents, different pH values, and different nitrate/reducing agent ratios, with the aim of improving the dispersion and further reducinh the size of nanoparticles. Different loadings of CeO_2_ will also be studied in order to maximize the performance while maintaining the lowest possible amount of active phase, due to the fact that cerium is considered to be a critical raw material, with the consequent necessity of drastically reducinh its use. Furthermore, the effect of different parameters (temperature, pressure, and MeOH/CO_2_ ratio) on the catalytic performance will be investigated.

## Figures and Tables

**Table 1:** Crystallite size (D_XRD_), BET surface area (S.A.), pore volume (V_p_), mean pore diameter (D_p_), mesostructure cell parameter (a_0_), and wall thickness (T_w_) of all the samples.

**Sample**	**D_XRD_ (nm)**	**a_0_ (nm)**	**S.A. (m^2^/g)**	**V_p_ (cm^3^/g)**	**D_P_ (nm)**	**T_w_ (nm)**
CeO_2__Meso	7.9 (1)	-	182	0.27	5.8	-
CeO_2_@SBA-15_TS	7.9 (1)	10.4	722	0.93	6.1	4.3
CeO_2_@SBA-15_SC	2.6 (1)	10.4	635	0.88	6.1	4.3
SBA-15	-	10.5	853	0.99	6.3	4.2

**Table 2:** Results of the catalytic tests. Conditions: 0.250 g of catalyst; 10 mL of liquid methanol; P = 5.0 MPa; T = 150 °C; reaction time = 3 h.

Catalyst	Yield (mmol/g_cat_)	Yield (mmol/g_act_._ph_.)	Yield (mol%)
CeO_2__Meso	0.941	0.941	2 × 10^−3^
CeO_2_@SBA-15_SC	0.097	0.971	2 × 10^−4^
CeO_2_@SBA-15_TS	0.066	0.662	1 × 10^−4^

## Data Availability

The original contributions presented in the study are included in the article/Supplementary Materials, further inquiries can be directed to the corresponding author.

## Supplementary Materials

The following supporting information can be downloaded at: https://www.mdpi.com/article/10.3390/nano14181490/s1, Figure S1: Rietveld refinement of CeO2_Meso; Figure S2: Rietveld refinement of CeO_2_@SBA-15_TS; Figure S3: Rietveld refinement of CeO_2_@SBA-15_SC; Figure S4: UV–Vis spectra of the samples; Figure S5: Thermogravimetric analysis of the samples; Figure S6: Additional line profile EDX analysis on CeO_2_@SBA-15_SC. The data for Ce, Si, and O have been normalized; Table S1: EDX quantitative analysis for CeO_2_@SBA15_TS; Table S2: EDX quantitative analysis for CeO_2_@SBA15_SC; References [67,68,69] are cited in the supplementary materials.
