# Supplementary material for: Ordered versus Non-Ordered Mesoporous CeO2-Based Systems for the Direct Synthesis of Dimethyl Carbonate from CO2
Source: Nanomaterials (Basel). 2024 Sep 13;14(18):1490. doi: 10.3390/nano14181490 (PMC11434316; doi:10.3390/nano14181490)
Supplement: Supplementary file 1 [file nanomaterials-14-01490-s001.zip › nanomaterials-3163430-supplementary.pdf]

Supplementary information

Ordered versus Non-Ordered Mesoporous CeO_2-Based Systems for the Direct Synthesis of Dimethyl Carbonate from CO_2

Nicoletta Rusta [1,2,†], Fausto Secci [1,2,†], Valentina Mameli [1,2] and Carla Cannas [1,2,*]

[1] Department of Chemical and Geological Sciences, University of Cagliari, S.S. 554 bivio per Sestu, 09042 Monserrato, CA, Italy; n.rusta@studenti.unica.it (N.R.); fausto.secci@unica.it (F.S.); valentina.mameli@unica.it (V.M.)

[2] Consorzio Interuniversitario Nazionale per la Scienza e Tecnologia dei Materiali (INSTM), Via Giuseppe Giusti 9, 50121 Firenze, FI, Italy

* Correspondence: ccannas@unica.it

† These authors contributed equally to this work.

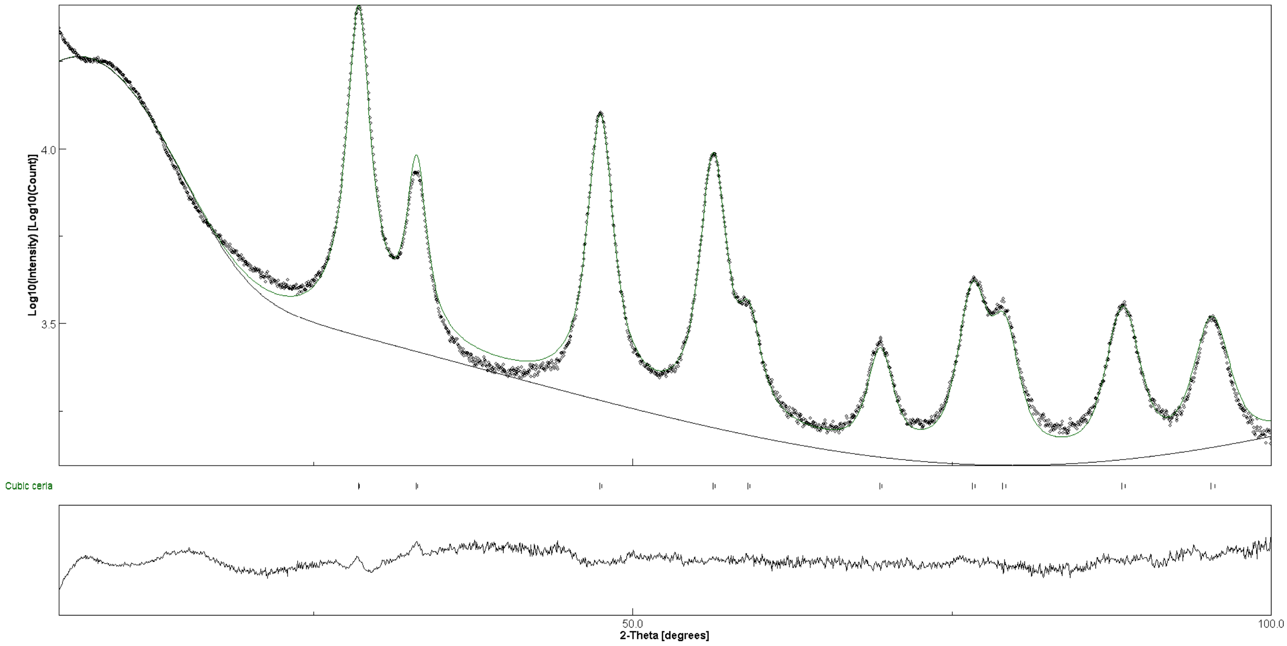

Figure S1 Rietveld refinement of CeO_2_Meso.

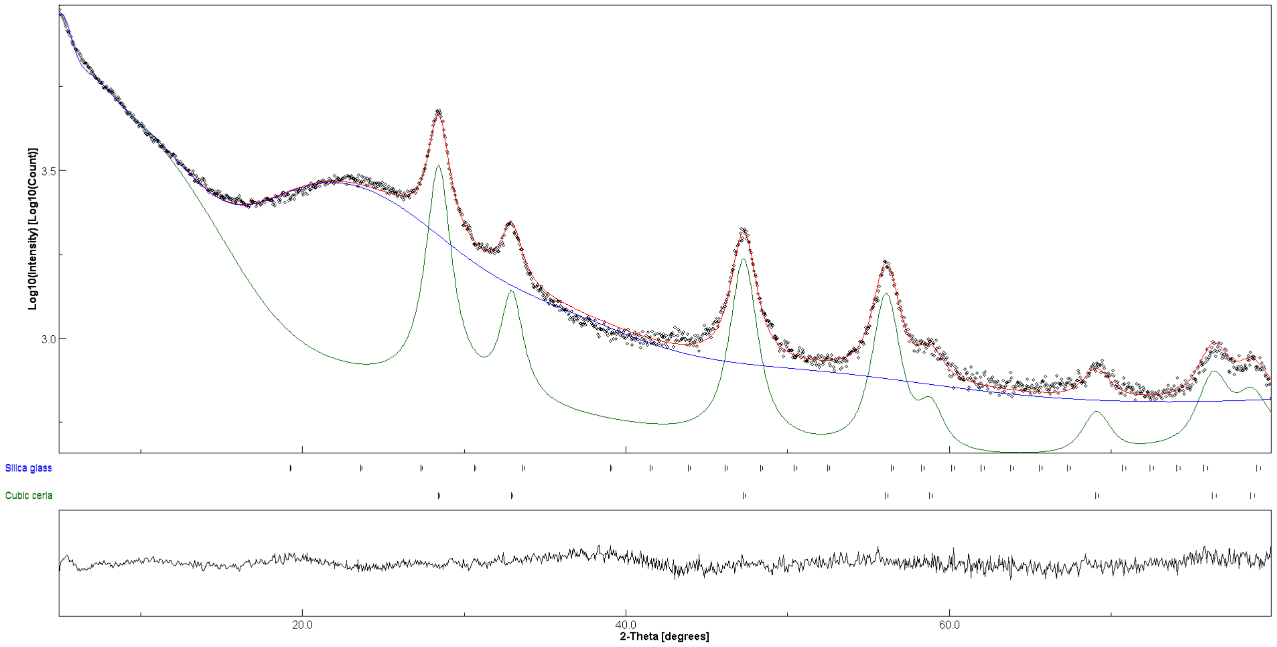

Figure S2 Rietveld refinement of CeO_2@SBA-15_TS.

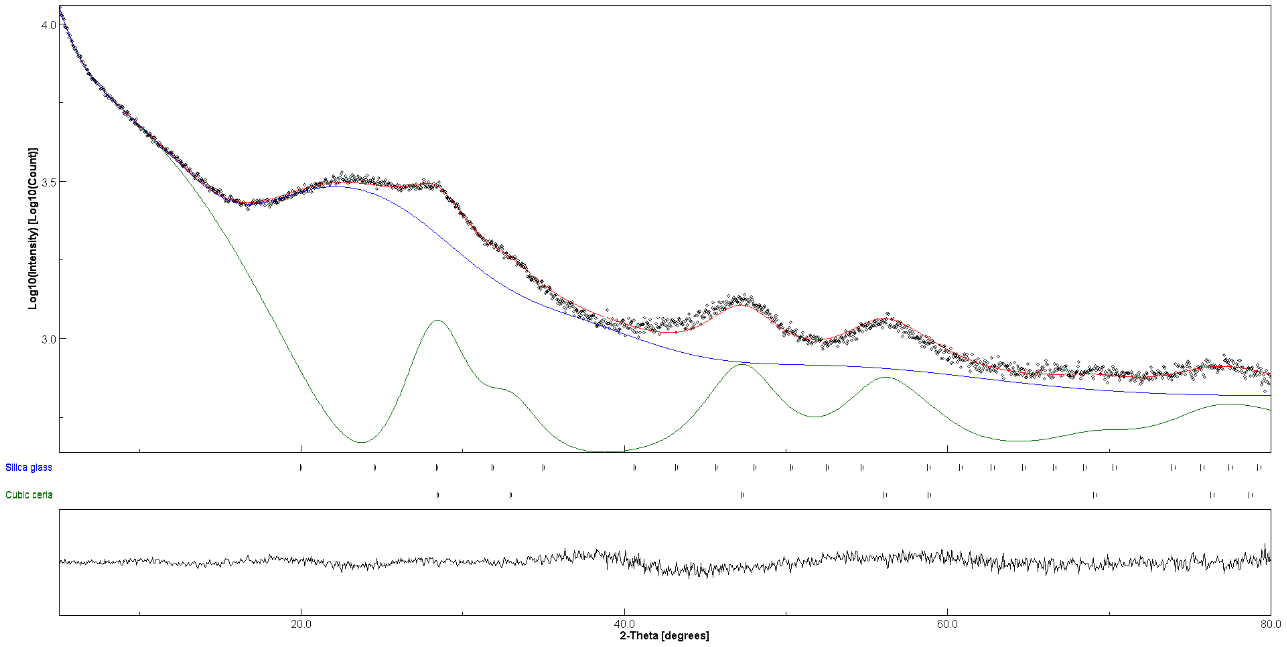

Figure S3 Rietveld refinement of CeO_2@SBA-15_SC.

Table S1 EDX quantitative analysis for CeO_2@SBA15_TS.

Spot	Element	Wt%	Wt% Sigma	Atomic %
Spot 1	Ce	7.75	0.52	1.13
	Si	33.82	0.45	24.51
	O	58.43	0.54	74.36
Spot 2	Ce	9.18	0.22	1.42
	Si	41.41	0.24	31.86
	O	49.41	0.26	66.73
Spot 3	Ce	8.71	0.23	1.33
	Si	40.99	0.26	31.28
	O	50.30	0.28	67.39
Spot 4	Ce	8.79	0.23	1.35
	Si	42.09	0.26	32.35
	O	49.12	0.28	66.29
Spot 5	Ce	9.89	0.31	1.55
	Si	42.43	0.33	33.12
	O	47.68	0.36	65.34

Table S2 EDX quantitative analysis for CeO_2@SBA15_SC.

Spot	Element	Wt%	Wt% Sigma	Atomic %
Spot 1	Ce	3.49	0.06	0.55
	Si	56.45	0.09	44.28
	O	40.06	0.09	55.17
Spot 2	Ce	2.56	0.12	0.38
	Si	48.04	0.18	35.52
	O	49.39	0.18	64.10
Spot 3	Ce	7.01	0.43	0.99
	Si	30.79	0.42	21.78
	O	62.20	0.50	77.23
Spot 4	Ce	12.78	0.30	1.98
	Si	35.06	0.28	27.14
	O	52.16	0.32	70.88
Spot 5	Ce	15.95	0.09	2.61
	Si	37.32	0.09	30.45
	O	46.73	0.10	66.94
Spot 6	Ce	3.13	0.34	0.43
	Si	31.99	0.30	21.83
	O	64.88	0.37	77.74
Spot 7	Ce	5.27	0.21	0.78
	Si	41.62	0.30	30.62
	O	53.11	0.31	68.60
Spot 8	Ce	6.34	0.26	0.95

	Si	42.99	0.33	32.27
	O	50.68	0.35	66.78
Spot 9	Ce	14.58	0.24	2.36
	Si	38.13	0.24	30.73
	O	47.29	0.27	66.91
Spot 10	Ce	15.87	0.25	2.64
	Si	40.31	0.24	33.47
	O	43.82	0.27	63.89
Spot 11	Ce	7.57	0.20	1.14
	Si	40.44	0.25	30.36
	O	51.99	0.27	68.50
Spot 12	Ce	14.15	0.19	2.29
	Si	39.17	0.19	31.60
	O	46.68	0.21	66.11
Spot 13	Ce	11.27	0.20	1.74
	Si	37.46	0.21	28.88
	O	51.27	0.24	69.38

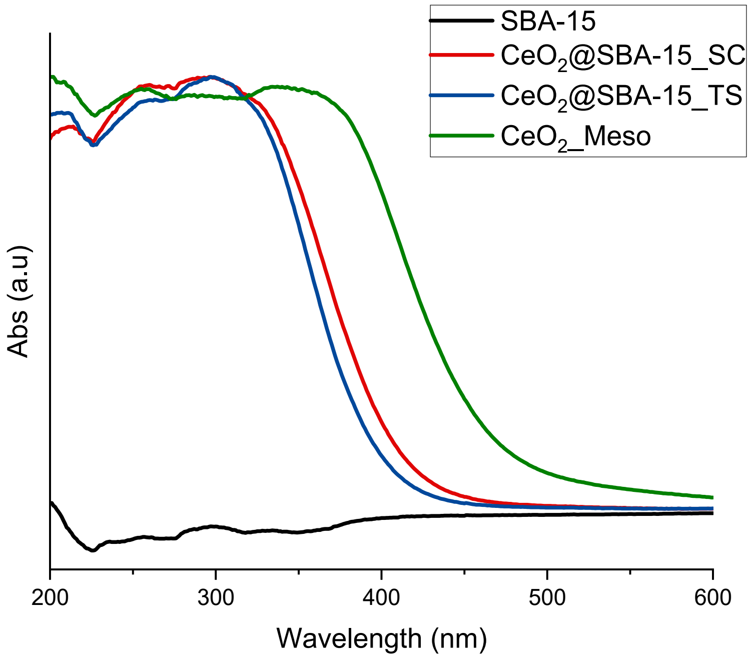

Figure S4 UV–Vis spectra of the samples.

Figure S4 shows the UV–Vis diffuse reflectance spectra (DRS) of the catalysts. The SBA-15 sample is characterized by an insignificant absorption due to its insulating properties, while the ceria samples show absorption in the UV–Vis region, as they exhibit semiconductor-like properties. The CeO_2-Meso sample shows a broad absorption band in the UV region below 500 nm composed of two or three contributions due to charge-transfer transitions from O 2p to Ce 4f, as observed by other authors for

mesoporous ceria samples [1,2]. The nanocomposites (CeO_2@SBA-15_SC and CeO_2@SBA-15_TS) present similar features to CeO_2-Meso, but the absorption contributions are shifted to lower wavelengths. The values of the contributions are compatible with those reported in the literature for similar CeO_2@SBA-15 samples synthesized by impregnation [3]. The small differences between the two nanocomposites may be attributable to the different impregnation strategy used and the consequent differences in ceria dispersion into the mesoporous channels and crystal size (XRD, TEM, and N_2-physisorption).

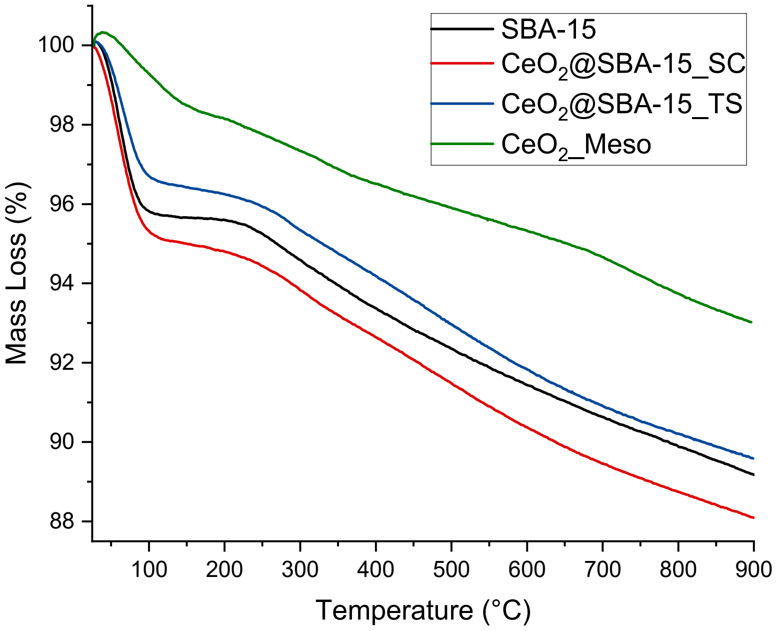

Figure S5 Thermogravimetric analysis of the samples.

Prior to the TGA characterization, the samples were treated at 120 °C overnight, like was performed prior to the catalytic tests. Despite these treatments, all samples show an initial weight loss steeper than the rest of the curve; this loss can be attributed to the desorption of water, probably adsorbed on the sample during the sample transfer and preparation procedure carried out before the analysis. This loss is much more evident for the two composites (3.4% for the TS composite and 4.8% for the SC composite) and their support (4.2%) compared to the CeO_2_Meso sample (1.5 %), likely due to their much higher surface area and pore volume, which allow for adsorbing a significantly larger amount of water. After the end of the water desorption branch, the CeO_2_Meso only shows a continuous and less steep weight decrease (7%) up to 900 °C, ascribable to the loss of the -OH surface groups; in particular, the Ce-OH groups are converted to Ce-O-Ce groups, releasing water. Regarding the three mesostructured samples, after the end of the water desorption, a plateau is observed for the SBA-15 support up to about 220 °C, indicating that no losses are present in this temperature range; the two composites, however, in the same 105–120 °C range, show a very shallow but not flat branch, due to the weight loss contribution from the CeO_2 active phase present on these samples. After 120 °C, the three mesostructured samples show a continuous and shallow loss of about 7% up to 900 °C, attributable to the loss of -OH surface groups, similarly to what was observed for the CeO_2_Meso sample. The TGA analysis results are reported in Figure S5.

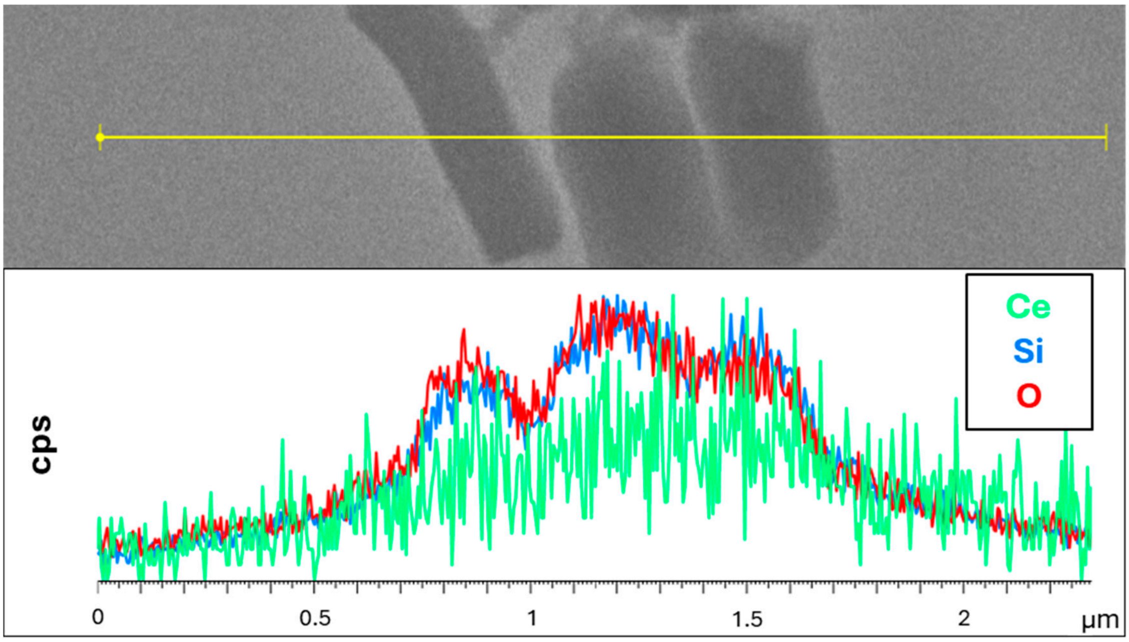

Figure S6 Additional line profile EDX analysis on CeO_2@SBA-15_SC. The data for Ce, Si, and O have been normalized.

References

1. Siranjeevi, R.; Vasumathi, V.; Suganya, S.; Saravanan, A.; Usha, R.; Azhagurajan, M.; Jeyalakshmi, R. Evaluation of Biosynthesized GO@CeO_2 Nanocomposites as a Catalyst for UV-Assisted Degradation of Organic Dyes and Phytotoxicity Studies. *Surfaces and Interfaces* 2024, *44*, 103748, doi:10.1016/j.surfin.2023.103748.

2. Meng, F.; Li, H.; Gong, J.; Fan, Z. Photocatalytic and Magnetic Properties of Loosened Ceria Hollow Microspheres Synthesized by a Single-Step Hydrothermal Method. *Journal of Materials Science: Materials in Electronics* 2016, *27*, 8433–8439, doi:10.1007/s10854-016-4857-y.

3. Mikheeva, N.N.; Zaikovskii, V.I.; Mamontov, G.V. Synthesis of Ceria Nanoparticles in Pores of SBA-15: Pore Size Effect and Influence of Citric Acid Addition. *Microporous and Mesoporous Materials* 2019, *277*, 10–16, doi:10.1016/j.micromeso.2018.10.013.
